# Constraining the brachial plexus does not compromise regional control in oropharyngeal carcinoma

**DOI:** 10.1186/1748-717X-8-173

**Published:** 2013-07-09

**Authors:** Mutter W Robert, Benjamin H Lok, Pinaki R Dutta, Nadeem Riaz, Jeremy Setton, Sean L Berry, Anuj Goenka, Zhigang Zhang, Shyam S Rao, Suzanne L Wolden, Nancy Y Lee

**Affiliations:** 1 Department of Radiation Oncology, Mayo Clinic, 200 First Street SW, Rochester, Minnesota 55905, USA; 2 Department of Radiation Oncology, Memorial Sloan-Kettering Cancer Center, New York, USA; 3 Department of Medical Physics, Memorial Sloan-Kettering Cancer Center, New York, USA; 4 Department of Epidemiology and Biostatistics, Memorial Sloan-Kettering Cancer Center, New York, USA

**Keywords:** Brachial plexopathy, Intensity-modulated radiation therapy, Oropharyngeal carcinoma, Cisplatin, Cetuximab

## Abstract

**Background:**

Accumulating evidence suggests that brachial plexopathy following head and neck cancer radiotherapy may be underreported and that this toxicity is associated with a dose–response. Our purpose was to determine whether the dose to the brachial plexus (BP) can be constrained, without compromising regional control.

**Methods:**

The radiation plans of 324 patients with oropharyngeal carcinoma (OPC) treated with intensity-modulated radiation therapy (IMRT) were reviewed. We identified 42 patients (13%) with gross nodal disease <1 cm from the BP. Normal tissue constraints included a maximum dose of 66 Gy and a D_05_ of 60 Gy for the BP. These criteria took precedence over planning target volume (PTV) coverage of nodal disease near the BP.

**Results:**

There was only one regional failure in the vicinity of the BP, salvaged with neck dissection (ND) and regional re-irradiation. There have been no reported episodes of brachial plexopathy to date.

**Conclusions:**

In combined-modality therapy, including ND as salvage, regional control did not appear to be compromised by constraining the dose to the BP. This approach may improve the therapeutic ratio by reducing the long-term risk of brachial plexopathy.

## Background

Brachial plexopathy is a potentially painful and debilitating complication of radiotherapy, characterized by sensory changes and motor deficits [1]. The time to the appearance of symptoms may vary significantly. Recent modern studies have reported median times to onset of symptoms ranging from 6.5 months to 4 years from the completion of radiotherapy and ranges of 1.4 months to 26 years [2,3]. Once they develop, there may be a gradual evolution of symptoms or a more rapid progression with time, which may on occasion culminate in complete loss of function of the affected arm [1,4]. Treatment options for these patients remain inadequate, and prognosis is poor. Brachial plexopathy has been associated with greater cumulative radiation dose to the brachial plexus (BP), radiotherapy fraction size, maximum dose, as well as the addition of chemotherapy and neck dissection [1,2,4-6]. One group recently used a validated symptom questionnaire in order to screen a large head-and-neck cancer population who had undergone radiation therapy. Their goal was to identify predictors of neuropathic symptoms thought to be related to brachial plexopathy. Interestingly, 12% of patients reported neuropathic symptoms, and there was a clear dose–response relationship [6]. They concluded that dose to the brachial plexus should be limited when possible. Suggested BP normal tissue constraints on recent IMRT head and neck protocols are shown in Table 1.

**Table 1:** Brachial plexus constraints on recent Radiation Therapy Oncology Group intensity-modulated radiotherapy head and neck cancer protocols

**Protocol**	**Brachial plexus constraint**
RTOG 0022	None specified
RTOG 0025	None specified
RTOG 0522	Dmax ≤60 Gy
RTOG 0615	Dmax ≤66 Gy*
RTOG 0619	Dmax ≤66 Gy, D05 ≤60 Gy
RTOG 0912	Dmax ≤66 Gy to point source at least 0.03 cm^3^**
RTOG 1008	Dmax <60 Gy if no involved low neck nodes; <66 Gy if low neck involved

RTOG 1016 None specified.

*The treating radiation oncologist has the option of prescribing an intermediate dose of 63 Gy, PTV_63_ in the lower neck close to the brachial plexus.

**Suggested dose limit that should not compromise tumor coverage.

Intensity-modulated radiotherapy (IMRT) allows for accurate targeting of disease while minimizing dose to surrounding critical normal structures and has become the standard treatment technique for the management of oropharyngeal carcinoma and many other head and neck cancers. When locally advanced head and neck cancers are treated definitively with IMRT, the planning target volume (PTV) encompassing gross primary and nodal disease is generally prescribed a dose of 70 Gy. If lymphadenopathy is present in proximity of the BP, however, it is often difficult to constrain the dose to the BP to meet normal tissue tolerance guidelines without at least some compromise of PTV coverage.

In the context of multi-modality therapy for head and neck cancers, including chemotherapy and neck dissection as salvage, IMRT treatment-planning guidelines were adopted at Memorial Sloan-Kettering Cancer Center in 2005 with the goal of reducing the risk of brachial plexopathy. These allowed for less than 100% coverage of the PTV in the low neck with the 70Gy isodose line to ensure adherence to BP normal tissue constraints of a maximum point dose (Dmax) of 65–66 Gy and a dose received by 5% of the tissue (D_05_) of 60 Gy. We recently reviewed the treatment plans of patients with squamous cell carcinoma of the oropharynx who underwent IMRT at our center. Our purpose was to determine whether these brachial plexus constraints impacted regional control.

## Materials and methods

### Patients/evaluation

Between January 2005 and April 2009, 324 patients with histologically confirmed squamous cell carcinoma of the oropharynx underwent definitive IMRT at our center. From this group of 324 patients definitively treated with IMRT after Institutional Review Board Approval we identified 42 patients (13%) who had clinically or radiographically positive gross nodal disease <1 cm from the BP. These 42 patients were the subjects of this study. January 2005 was chosen as a cut-off because it was at that time that we began contouring and constraining the BP for all patients treated with IMRT for head and neck cancer at our institution. This retrospective analysis was approved by our Institutional Review Board.

Pretreatment evaluation included a complete history and physical examination, flexible fiberoptic endoscopic examination, complete blood counts, liver function tests, chest X-ray, and dental evaluation, as well as magnetic resonance imaging and/or computed tomography (CT) scans of the head-and-neck region. CT scans of the chest and abdomen, and positron emission tomography (PET) scans were also obtained for most patients before the start of treatment.

### Treatment

Our treatment-planning techniques have previously been described [7-9]. To account for setup error, the PTV_70_ was defined to encompass the gross primary and neck disease plus margin (0.3-0.5 cm), the PTV_59.4_ was defined as the high-risk subclinical disease plus margin (0.3-0.5 cm), and the PTV_54_ was defined as the low-risk subclinical disease plus margin (0.3 cm). We frequently use a split-field technique for cancers of the oropharynx when lymph nodes are not situated near the larynx, in order to reduce the dose to that structure. All 42 patients reported here, however, had clinically a dose-painting whole-neck IMRT technique [8].

Platinum-based chemotherapy was administered to 31 of the 42 patients (74%). Concurrent single-agent cisplatin was the primary choice of chemotherapeutic agent, consisting of a planned two to three cycles (100 mg/m^2^) of cisplatin on days 1, 22, and 43. Eleven (26%) received cetuximab. Routine planned neck dissections were not undertaken. Rather, a PET/CT scan was typically performed 3 months after the completion of radiotherapy and neck dissection was reserved for patients with less than a complete response on imaging or clinical examination. Location of dissected lymph nodes was determined prospectively based on pathology reports.

### Constraining the brachial plexus

The BP was contoured for all patients prior to treatment using a modified version of the Radiation Therapy Oncology Group guidelines, with the most superior extent of the structure at the level of the cricoid cartilage. Normal tissue constraints adhered to included a Dmax of 66 Gy and a D_05_ of 60 Gy for the BP. Gross tumor volume coverage was generally prioritized. However, the BP dose volume criteria routinely took precedence over PTV coverage near the BP. In order to study the dosimetric impact of the BP constraints on PTV coverage, we defined PTV_BP70_ as the portion of the PTV prescribed 70 Gy from 6 mm superior to 6 mm inferior of the ipsilateral BP. The 6 mm distance was chosen because PTV coverage outside of this range would not be expected to be significantly impacted by the BP avoidance structure. Any recurrences occurring within or adjacent to the PTV_BP70_ were presumed to have been potentially caused by the BP dose constraint. Dose volume histograms were created for all 42 patients and dose volume parameters for the BP and PTV_BP70_ were analyzed. Thirteen patients had bilateral gross nodal disease within 1 cm of the BP. The dose volume parameters for both the BP and PTV_BP70_ were analyzed independently in these patients.

### Follow-up

Patients were evaluated weekly during RT. After the completion of radiation, patients were evaluated every 2–3 months for the first 2 years and every 4–6 months thereafter. All All patients, including those who developed distant metastases, continued to be monitored closely with regular physical exams, flexible fiberoptic endoscopy, and radiographical studies to assess for locoregional recurrence and symptoms of brachial plexopathy. Human papillomavirus (HPV) status was unavailable for patients on this study, but smoking status, including median number of pack-years, is reported.

### Statistical methods

The cumulative incidence function was used to describe local recurrence, regional failure, and distant metastasis. Death without recurrence was regarded as a competing risk [10]. The time to recurrence was defined as the time (in months) elapsed from the start of radiation to the date of recurrence, death, or last follow-up. The Kaplan-Meier method was used to describe overall survival [11].

## Results

Characteristics of the 42 patients are summarized in Table 2. The primary site was located in the base of tongue in 69% and in the tonsil in 31%. Seventy-one percent of patients were greater than 55 years old. Patients in this study had low neck disease involvement in proximity of the BP, and therefore relatively advanced-stage disease compared to all patients presenting with oropharyngeal carcinoma [12,13]. Forty-one of 42 patients (97.6%) were AJCC stage IV, including 6 patients (14.3%) with lymph nodes greater than 6 cm in maximum diameter (N3).

**Table 2:** Patient characteristics

**Characteristic**	**Number (%)**
Sex	
Male	39 (92.9%)
Female	3 (7.1%)
Age	
≤55	12 (28.6%)
>55	30 (71.4%)
Race	
White	37 (88.1%)
Not white	5 (11.9%)
KPS	
90-100	34 (81.0%)
60-80	8 (19.0%)
Primary site	
Tonsil	13 (31.0%)
Base of tongue	29 (69.0%)
Pharyngeal wall	0 (0%)
Soft palate	0 (0%)
T stage	
T1	7 (16.7%)
T2	21 (50.0%)
T3	6 (14.3%)
T4	8 (19.0%)
N stage	
N0	0 (0%)
N1	2 (4.8%)
N2	34 (81.0%)
N3	6 (14.3%)
AJCC Stage	
I	0 (0%)
II	0 (0%)
III	1 (2.4%)
IV	41 (97.6%)
Tobacco exposure	
Never smoked	8 (19.0%)
Former smoker	26 (61.9%)
Current smoker	7 (16.7%)
Number of pack years	
Median (range)	8 (0–84)
Neck dissection before radiation	
No	42 (100%)
Yes	0 (0%)
Chemotherapy	42 (100%)
Concurrent	38 (90.5%)
Cisplatin	20 (47.6%)
Carboplatin/5-FU	1 (2.4%)
Carboplatin/paclitaxel	1 (2.4%)
Cetuximab	11 (26.2%)
Cisplatin/bevacizumab	5 (11.9%)
Induction + concurrent	3 (7.1%)

Abbreviations: AJCC American Joint Committee on Cancer, 5-FU 5-fluorouracil, KPS Karnofsky performance status.

**Figure 1:**
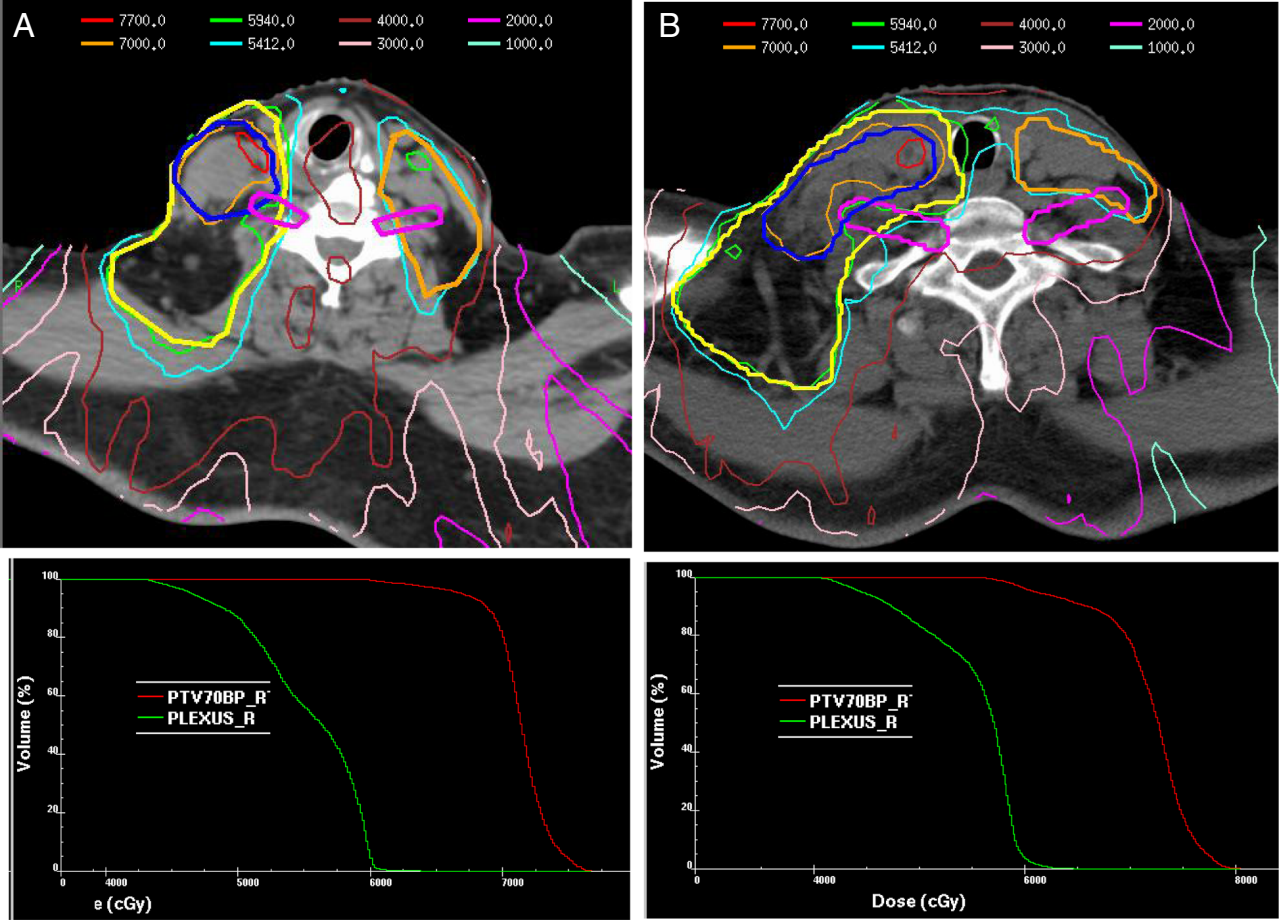
A. and B. CT slices through the low neck at the level of the brachial plexus along with corresponding dose-volume histograms in two representative patients demonstrating constraining of the brachial plexus at the expense of the immediately adjacent gross nodal disease PTV coverage.

Dose volume parameters of the BP and PTV_BP70_ for the entire cohort are displayed in Table 3. For the PTV_BP70_, the median D95 was 66.7 Gy and the median minimum point dose (Dmin) was 58.7 Gy. For the BP, the median Dmax was 63.4 Gy and the median D05 was 59.1 Gy. Just one patient in this study had both a BP Dmax and D_05_ greater than 65 Gy and 60 Gy, respectively. The radiation plans with corresponding isodose lines for two representative patients are displayed in Figure 1A and Figure 1B.

**Table 3:** Dose volume parameters

**Parameter**	**Median dose Gy (SD)**
PTVBP70	
D95	66.7 (6.4)
Minimum dose	58.7 (8.5)
Mean dose	71.9 (4.0)
D05	75.2 (5.1)
Maximum dose	76.9 (2.5)
Brachial plexus	
Maximum dose	63.4 (4.1)
D05	59.1 (4.8)
Mean dose	52.8 (11.9)

### Treatment outcomes

The median follow-up time was 35 months (range, 1–69). Of the 42 patients, 8 underwent post-RT neck dissection due to clinical or radiograph evidence of residual disease following RT. For the PTV_BP70_ of these 8 patients, the median D95 was 67.4 Gy and the median Dmin was 59.4 Gy. Histologic evidence of residual cancer was identified in 4 of these 8 patients. In one of the 4 patients, a single level 2 node contained viable tumor. In three patients, multiple lymph nodes from the upper as well as the lower neck contained viable tumor.

**Figure 2:**
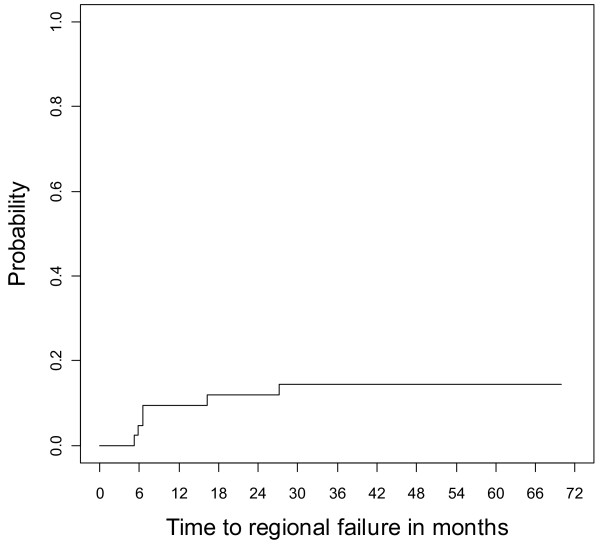
Cumulative incidence of regional recurrence.

To date, there have been six regional recurrences. The cumulative incidence of regional failure was 14% (95% CI 0.04- 0.25, Figure 2). For the PTV_BP70_ of these 6 patients, the median D95 was 67.96 Gy and the median Dmin was 59.2 Gy, demonstrating slightly greater PTV coverage than the median for the entire cohort. Review of pre- and post-treatment imaging, operative notes, and pathology reports demonstrated that just one isolated regional failure occurred close to the PTV_BP70_ This patient presented with a >10 cm level 3 node invading the skin. Following chemoradiation, there was residual hypermetabolic activity in the periphery of the mass, and a planned neck dissection was performed. Pathology demonstrated a cystic nodule of squamous cell carcinoma that was mostly necrotic (<1% viable tumor cells present). All 28 other excised lymph nodes were negative. There was concern at surgery for possible unresectable residual disease at the carotid bulb which in this patient was above the brachial plexus at the level of the 4th cervical vertebral body. Therefore regional re-irradiation was administered postoperatively. The patient developed evidence of distant metastases 2 months following the completion of re-irradiation and eventually succumbed from his disease with disease locoregionally controlled. The D_95_ and Dmin of the PTV_BP70_ for that patient were 62.3 and 51.6 Gy, respectively. The other 5 regional failures in the study included: Patient 1) A new contralateral out-of-field level 5 nodal neck failure in a previously node-negative hemi-neck; Patient 2) A new ipsilateral high neck lymph node at level II; Patients 3 and 4) Local recurrences with an adjacent regional node in the high level II neck; 5) Local recurrence and regional disease in level II and level IV.

The median number of pack-years smoked for the 6 patients with regional failures was 13. One of the six patients was a never smoker, and 4 had a greater than 10 pack-year smoking history. Due to the small number of events, we could not determine whether smoking status was associated with a higher likelihood of regional recurrence.

**Figure 3:**
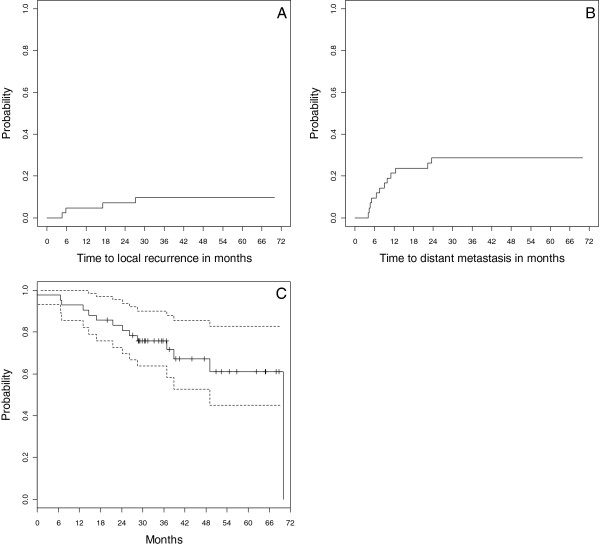
Cumulative incidence of local recurrence (A), distant metastasis (B), and Kaplan-Meier estimates of overall survival (C).

The cumulative incidence of local recurrence and distant metastasis were 10% (95% confidence interval [CI] 0.005-0.19, Figure 3A), and 29% (95% CI 0.15-0.43, Figure 3B), respectively. The 3-year Kaplan-Meier estimated overall survival probability was 76% (95% CI 0.64-0.90, Figure 3C). There have been no reported episodes of brachial plexopathy to date.

## Discussion

To reduce the risk of the potentially debilitating late effect of brachial plexopathy, BP dose constraints for head and neck cancer IMRT treatment planning were adopted at our institution that took precedence over PTV coverage in the vicinity of the BP. The cohort of patients with oropharayngeal squamous cell carcinoma examined here all had lymphadenopathy extending into levels III or IV of the neck, where there was risk of overlap with the brachial plexus. Therefore, they had more advanced disease and more adverse prognostic features than seen in recent reports and clinical trials for this disease [9,12,14]. For example, the rates of local failure and distant metastases in our updated experience treating patients with oropharyngeal carcinoma with IMRT were 5.4% and 12.5%, compared to 10% and 29% in the subset reported here [13]. Despite these adverse features, just one patient experienced an isolated regional recurrence in proximity of the BP. This patient had a level III node >10 cm in maximal diameter, invading into the skin. At the time of neck dissection for residual tumor, there was concern of persistent disease at the carotid bulb which was not amenable to resection. The carotid bulb is well above the C5 nerve root of the BP. Therefore, it is unlikely that the BP constraint had an impact on this treatment outcome, although we cannot exclude that possibility. Ultimately, local control was established with a course of postoperative re-irradiation. Four other patients had regional recurrences on the ipsilateral side of the constrained brachial plexus, but as described above, these tended to be in the high neck and associated with local recurrence at the primary. These patterns of recurrence suggest that tumor biology, rather than brachial plexus dose constraints, were likely responsible for the clinical outcomes and that the treatment approach we adopted, aimed at reducing the long-term risk of brachial plexopathy, may ultimately improve the therapeutic ratio.

Indeed, all 324 patients treated with IMRT for oropharyngeal carcinoma at our institution were spared high doses to the BP during the time period of this study. This is particularly important as recent evidence has emerged, more clearly establishing a dose–response relationship for the development of brachial plexopathy. One report in head and neck cancer patients following RT suggested that the incidence of patient-reported BP-associated neuropathies may be as high as 22% with long-term follow-up. In that study, Chen et al. demonstrated that radiation maximum dose to the BP was predictive of symptoms. They noted a significantly higher number of patients with symptoms who received doses of >70 Gy, suggesting a threshold effect [2,6]. Moreover, each Gy increase in maximum dose was associated with 1.39 times greater odds of developing symptoms. In another modern study of lung cancer patients treated with definitive chemoradiation, receipt of a median brachial plexus dose of >69 Gy and a maximum dose of >75 Gy to 2 cm^3^ were independent predictors of brachial plexopathy.

The median follow-up time in our study was 35 months; therefore, more time is required to determine the true long-term risk of brachial plexopathy for this cohort of patients where careful attention was paid to limiting the dose to the BP [6]. Moreover, it is possible that a more sensitive screening tool may better identify patients with neuropathic symptoms that could potentially be attributable to RT. To date, however, it is re-assuring that by limiting a small volume of the BP to doses approaching 65–66 Gy, the incidence of brachial plexopathy in our patients, closely followed in a multimodality setting, has been low. This finding is consistent with these studies as well as consensus guidelines [2,6,15].

The clinical decision balancing adequate PTV coverage in the low neck with risk of brachial plexopathy is commonly encountered in radiation oncology practice, not unlike other clinical sites such as the central nervous system, where protection of organs at risk such as the optic chiasm or brainstem may impact PTV coverage. Over 10% of all patients that presented to our institution who underwent definitive radiation for oropharyngeal squamous cell carcinoma had gross disease less than 1 cm from the BP. In another study, an estimated 20% of patients with head and neck cancer treated with IMRT had BP maximum point doses of >66 Gy [16]. In addition, there is accumulating evidence of a rise in incidence of oropharyngeal cancer in the United States and Western Europe that is associated with an increase in human papillomavirus (HPV)-associated cancers [17,18]. HPV-related oropharyngeal carcinomas are associated with younger age at diagnosis [4,14]. Moreover, HPV positivity is an independent predictor of improved overall survival [14,19]. Therefore, with patients experiencing longer overall survival rates in this new era of viral-induced carcinoma, efforts to reduce the dose to normal tissue structures, like the brachial plexus, will be of greater importance to limit risks of brachial plexopathy and other late effects of therapy.

One limitation of our study is the lack of tumor HPV status, which has been shown to be associated with improved progression-free and overall survival in oropharyngeal squamous cell carcinoma [14,20]. We did, however, have data on tobacco smoking history, which is inversely correlated with HPV status and has also been independently associated with progression-free and overall survival. Due to the small number of recurrences, however, we are unable to draw meaningful conclusions on the impact of smoking status on regional control, let alone control of disease in the vicinity of the BP. We presently continue to apply the same BP constraints for both HPV positive and and HPV negative patients seen in our clinic.

At the Radiation Therapy Oncology Group (RTOG), BP constraints have recently been incorporated into IMRT head and neck cancer protocols (Table 1). Nevertheless, there remains some variation among protocols and controversy about the appropriate treatment approach when disease involves the low neck. For example, on RTOG 0615 for nasopharyngeal carcinoma, the BP dose constraint is a maximum dose ≤66 Gy. On that protocol, the treating radiation oncologist has the option of prescribing an intermediate dose of 63 Gy in the lower neck close to the BP [21]. For RTOG 0912 for anaplastic thyroid carcinoma, BP constraints include a BP maximum dose ≤66 Gy to a point source at least 0.03 cm^3^. However, RTOG 0912 specifies that this “suggested dose limit” should not compromise tumor coverage [22]. This is most likely due to the low life expectancy of anaplastic thyroid cancer. For RTOG 1016, the phase 3 trial of radiotherapy plus cetuximab versus chemoradiotherapy in HPV-associated oropharynx cancer, the BP is not defined as a normal tissue/organ at risk [23]. Notably, the dose specification for low neck nodes on that study is 60 Gy in 30 fractions. Our results may provide some re-assurance to clinicians enrolling patients on these and future studies that BP constraints similar to those used in our study, will not be expected to impact regional control.

It is noteworthy that no patients underwent adaptive radiotherapy with replanning during the course of treatment to account for anatomical changes, such as a reduction in volume of neck lymphadenopathy. It is possible that, in some patients, the dose to the PTV in proximity of the BP was actually greater than what was calculated from the original treatment plan as a result of shrinkage of gross disease away from the BP and into the higher-dose region of the PTV. In addition, all patients in this study received concurrent chemoradiation, the majority of which was platinum based. Cisplatin has been perhaps the most widely used chemotherapeutic agent in combination with radiation. The biological basis of radiation sensitization from platinum is evolving but the most widely believed explanation is through inhibition of sublethal damage repair [24]. We have previously reported inferior locoregional control with concurrent cetuximab-based chemoradiation compared with cisplatin [25]. We would therefore caution that our data may not imply the safety of any compromise of PTV coverage to meet BP dose constraints in patients not receiving concurrent cisplatin-based chemotherapy.

## Conclusion

In conclusion, in the context of combined-modality therapy, including ND as salvage, regional control did not appear to be compromised by constraining the dose to the BP. We expect that attention to these normal tissue dose-volume parameters will ultimately reduce the risk of brachial plexopathy long-term. Our findings suggest that when multimodality therapy is utilized to treat OPC, BP constraints resembling those adopted at our institution are safe. Furthermore, our study provides data to justify constraining the BP in present and future IMRT protocols for OPC. Close attention to sites of recurrence are warranted on these prospective studies, however, to confirm the findings reported here.

## Competing interests

The authors declare that they have no competing interest.

## Authors’ contributions

NL, RM, PD, and BL conceived of the study and participated in its design and coordination. RM, BL, PD, NR, JS, SB, AG, SR, SW, and NL accumulated and analyzed data. ZZ performed statistical analysis. SB, BL, and RM carried out the dosimetric analysis. RM and NL drafted the manuscript. All authors read and approved the final manuscript.

## References

[B1] Schierle C Winograd JM Radiation-induced brachial plexopathy: review. Complication without a cure. J Reconstr Microsurg 2004 20 149 152 15011123 10.1055/s-2004-820771

[B2] Amini A Yang J Williamson R McBurney ML Erasmus J Jr Allen PK Karhade M Komaki R Liao Z Gomez D Cox J Dong L Welsh J Dose constraints to prevent radiation-induced brachial plexopathy in patients treated for lung cancer Int J Radiat Oncol Biol Phys 2012 82 e391 e398 10.1016/j.ijrobp.2011.06.1961 22284035 PMC3786565

[B3] Kori SH Foley KM Posner JB Brachial plexus lesions in patients with cancer: 100 cases Neurology 1981 31 45 50 10.1212/WNL.31.1.45 6256684

[B4] Powell S Cooke J Parsons C Radiation-induced brachial plexus injury: follow-up of two different fractionation schedules Radiother Oncol 1990 18 213 220 10.1016/0167-8140(90)90057-4 2217869

[B5] Svensson H Westling P Larsson LG Radiation-induced lesions of the brachial plexus correlated to the dose-time-fraction schedule Acta Radiol Ther Phys Biol 1975 14 228 238 10.3109/02841867509132663 1163290

[B6] Chen AM Hall WH Li J Beckett L Farwell DG Lau DH Purdy JA Brachial plexus-associated neuropathy after high-dose radiation therapy for head-and-neck cancer Int J Radiat Oncol Biol Phys 2012 84 165 169 10.1016/j.ijrobp.2011.11.019 22444998

[B7] De Arruda FF Puri DR Zhung J Narayana A Wolden S Hunt M Stambuk H Pfister D Kraus D Shaha A Shah J Lee NY Intensity-modulated radiation therapy for the treatment of oropharyngeal carcinoma: the memorial sloan-kettering cancer center experience Int J Radiat Oncol Biol Phys 2006 64 363 373 10.1016/j.ijrobp.2005.03.006 15925451

[B8] Lee N Mechalakos J Puri DR Hunt M Choosing an intensity-modulated radiation therapy technique in the treatment of head-and-neck cancer Int J Radiat Oncol Biol Phys 2007 68 1299 1309 10.1016/j.ijrobp.2006.11.019 17241750

[B9] Lok BH Setton J Caria N Romanyshyn J Wolden SL Zelefsky MJ Park J Rowan N Sherman EJ Fury MG Ho A Pfister DG Wong RJ Shah JP Kraus DH Zhang Z Schupak KD Gelblum DY Rao SD Lee NY Intensity-modulated radiation therapy in oropharyngeal carcinoma: effect of tumor volume on clinical outcomes Int J Radiat Oncol Biol Phys 2012 82 1851 1857 10.1016/j.ijrobp.2011.03.029 21640497 PMC4978948

[B10] Gray R A class of K-sample tests for comparing the cumulative incidence of a competing risk Ann Stat 1988 16 1141 1154 10.1214/aos/1176350951

[B11] Kaplan E Meier P Nonparametric-estimation from incomplete observations J Am Stat Assoc 1958 53 457 481 10.1080/01621459.1958.10501452

[B12] Denis F Garaud P Bardet E Alfonsi M Sire C Germain T Bergerot P Rhein B Tortochaux J Calais G Final results of the 94–01 french head and neck oncology and radiotherapy group randomized trial comparing radiotherapy alone with concomitant radiochemotherapy in advanced-stage oropharynx carcinoma J Clin Oncol 2004 22 69 76 14657228 10.1200/JCO.2004.08.021

[B13] Setton J Caria N Romanyshyn J Koutcher L Wolden SL Zelefsky MJ Rowan N Sherman EJ Fury MG Pfister DG Wong RJ Shah JP Kraus DH Shi W Zhang Z Schupak KD Gelblum DY Rao SD Lee NY Intensity-modulated radiotherapy in the treatment of oropharyngeal cancer: an update of the memorial sloan-kettering cancer center experience Int J Radiat Oncol Biol Phys 2012 82 291 298 10.1016/j.ijrobp.2010.10.041 21167652

[B14] Ang KK Harris J Wheeler R Weber R Rosenthal DI Nguyen-Tan PF Westra WH Chung CH Jordan RC Lu C Kim H Axelrod R Silverman CC Redmond KP Gillison ML Human papillomavirus and survival of patients with oropharyngeal cancer N Eng J Med 2010 363 24 35 10.1056/NEJMoa0912217 PMC2943767 20530316

[B15] Emami B Lyman J Brown A Coia L Goitein M Munzenrider JE Shank B Solin LJ Wesson M Tolerance of normal tissue to therapeutic irradiation Int J Radiat Oncol Biol Phys 1991 21 109 122 2032882 10.1016/0360-3016(91)90171-y

[B16] Truong MT Romesser PB Qureshi MM Kovalchuk N Orlina L Willins J Radiation dose to the brachial plexus in head-and-neck intensity-modulated radiation therapy and its relationship to tumor and nodal stage Int J Radiat Oncol Biol Phys 2012 84 158 164 10.1016/j.ijrobp.2011.10.079 22300574 PMC5014352

[B17] Chaturvedi AK Engels EA Anderson WF Gillison ML Incidence trends for human papillomavirus-related and -unrelated oral squamous cell carcinomas in the united states J Clin Oncol 2008 26 612 619 10.1200/JCO.2007.14.1713 18235120

[B18] Nasman A Attner P Hammarstedt L Du J Eriksson M Giraud G Ahrlund-Richter S Marklund L Romanitan M Lindquist D Ramqvist T Lindholm J Sparen P Ye W Dahlstrand H Munck-Wikland E Dalianis T Incidence of human papillomavirus (HPV) positive tonsillar carcinoma in stockholm, sweden: an epidemic of viral-induced carcinoma? International journal of cancer. Journal international du cancer 2009 125 362 366 10.1002/ijc.24339 19330833

[B19] Fakhry C Westra WH Li S Cmelak A Ridge JA Pinto H Forastiere A Gillison ML Improved survival of patients with human papillomavirus-positive head and neck squamous cell carcinoma in a prospective clinical trial J Natl Cancer Inst 2008 100 261 269 10.1093/jnci/djn011 18270337

[B20] Kong FM Ritter T Quint DJ Senan S Gaspar LE Komaki RU Hurkmans CW Timmerman R Bezjak A Bradley JD Movsas B Marsh L Okunieff P Choy H Curran WJ Jr Consideration of dose limits for organs at risk of thoracic radiotherapy: atlas for lung, proximal bronchial tree, esophagus, spinal cord, ribs, and brachial plexus Int J Radiat Oncol Biol Phys 2011 81 1442 1457 10.1016/j.ijrobp.2010.07.1977 20934273 PMC3933280

[B21] Radiation Therapy Oncology Group (RTOG) 0615 A phase II study of concurrent chemoradiotherapy using three-dimensional conformal radiotherapy (3D-CRT) or intensity-modulated radiation therapy (IMRT) + bevacizub (BV) for locally or regionally advanced nasopharyngeal cancer 2011 [http://www.rtog.org/ClinicalTrials/ProtocolTable/StudyDetails.aspx?study=0615. Accessed Nov. 11, 2011]23884282

[B22] Radiation Therapy Oncology Group (RTOG) 0912 A randomized phase II study of concurrent intensity modulated radiation therapy (IMRT), paclitaxel and pazopanib (NSC 737754)/placebo, for the treatment of anaplastic thyroid cancer 2011 http://www.rtog.org/ClinicalTrials/ProtocolTable/StudyDetails.aspx?study=0912. Accessed Nov. 11, 2011

[B23] Radiation Therapy Oncology Group (RTOG) 1016 2011 http://www.rtog.org/ClinicalTrials/ProtocolTable/StudyDetails.aspx?study=1016. Accessed Nov. 11, 2011 8750473

[B24] Wilson GD Bentzen SM Harari PM Biologic basis for combining drugs with radiation Semin Radiat Oncol 2006 16 2 9 10.1016/j.semradonc.2005.08.001 16378901

[B25] Koutcher L Sherman E Fury M Wolden S Zhang Z Mo Q Stewart L Schupak K Gelblum D Wong R Kraus D Shah J Zelefsky M Pfister D Lee N Concurrent cisplatin and radiation versus cetuximab and radiation for locally advanced head-and-neck cancer Int J Radiat Oncol Biol Phys 2011 81 915 922 10.1016/j.ijrobp.2010.07.008 20947269

